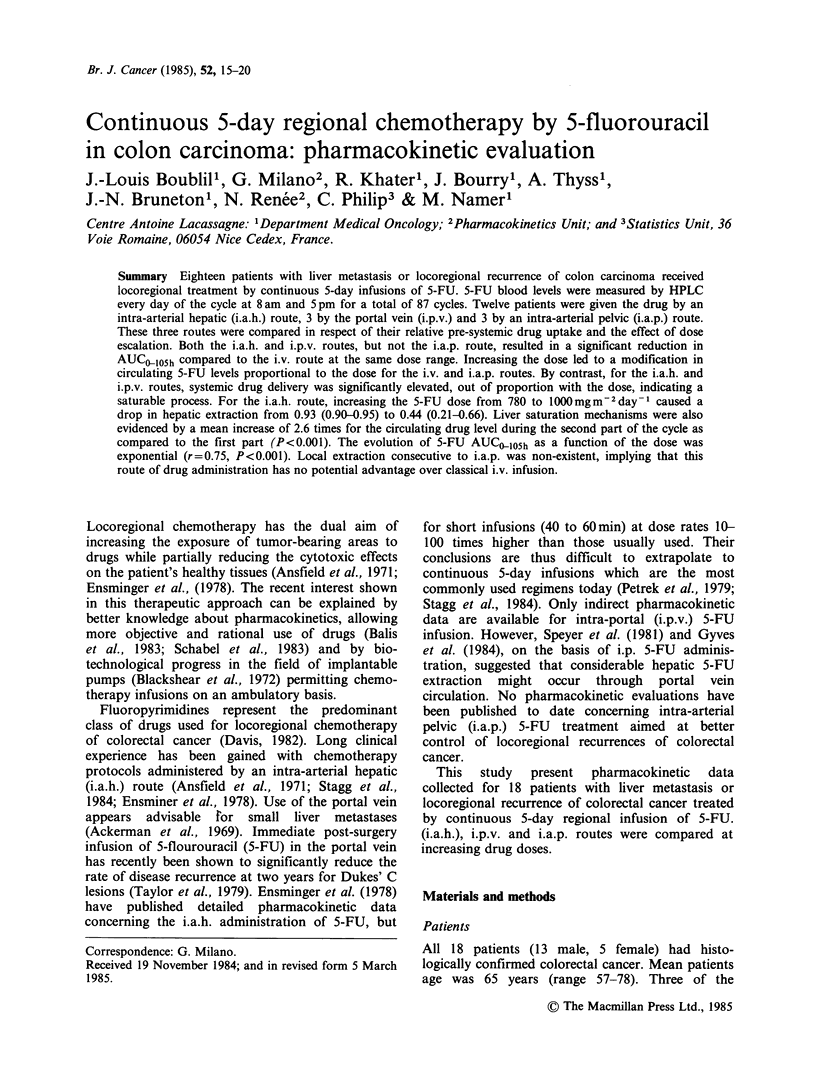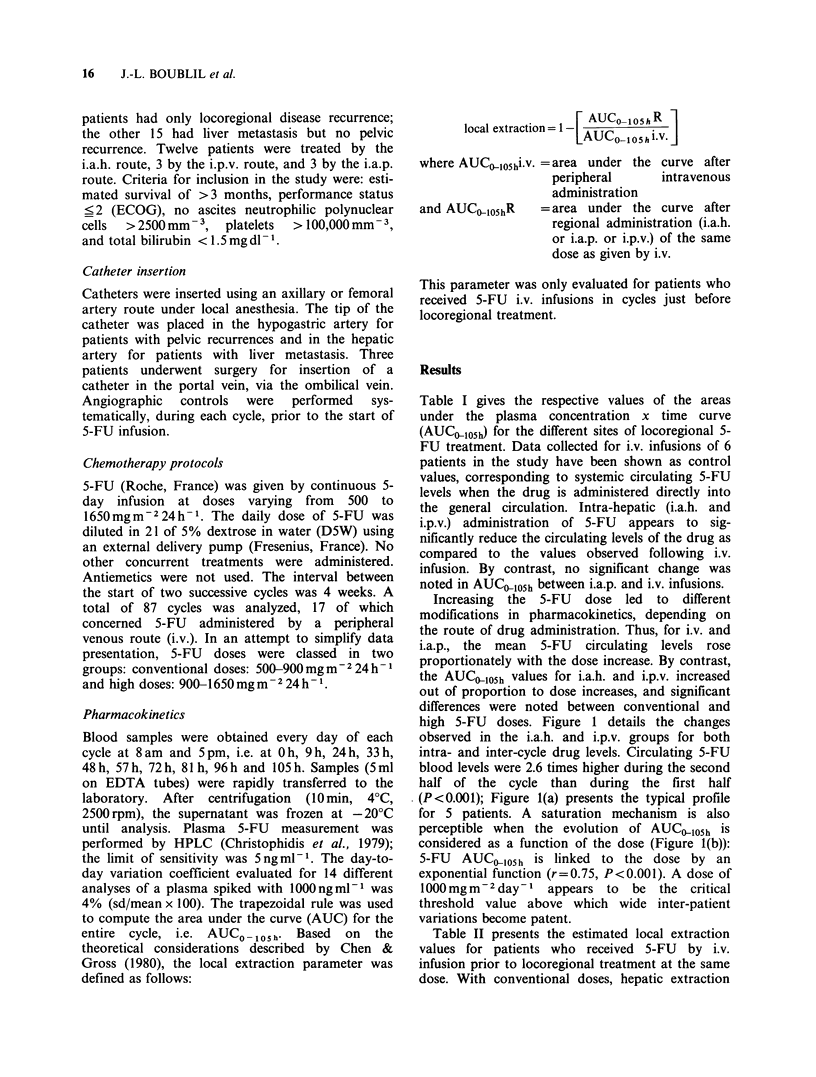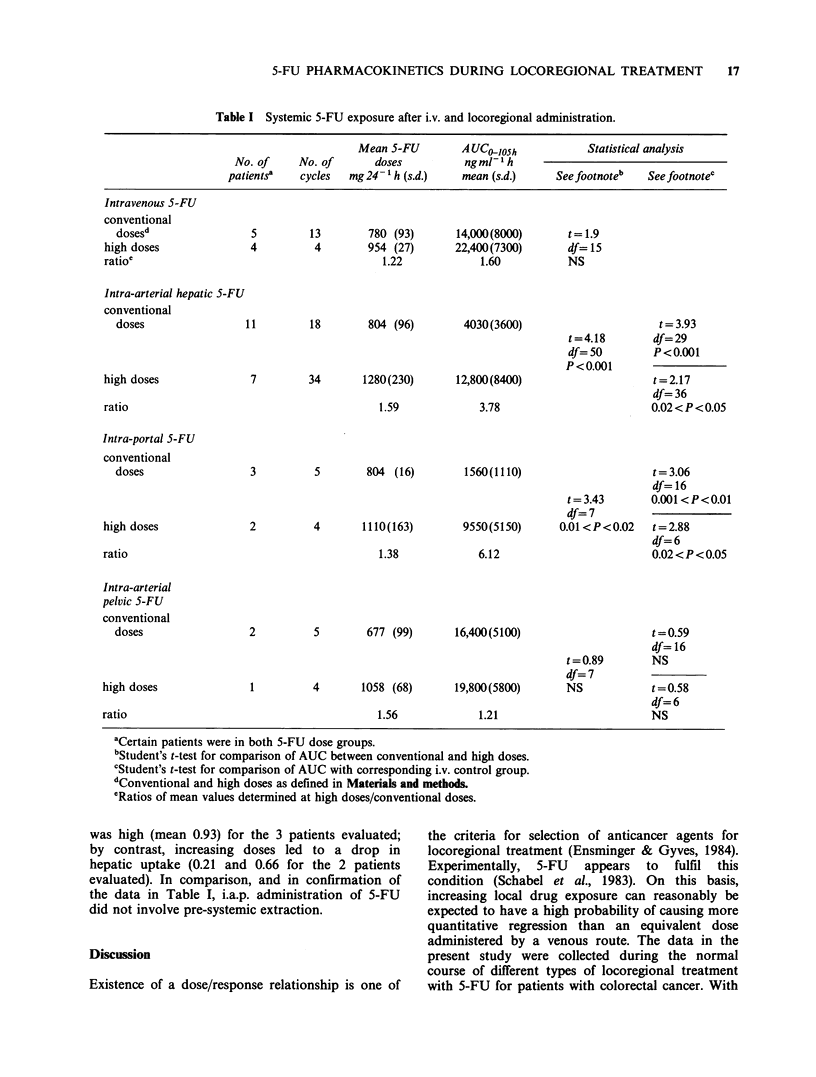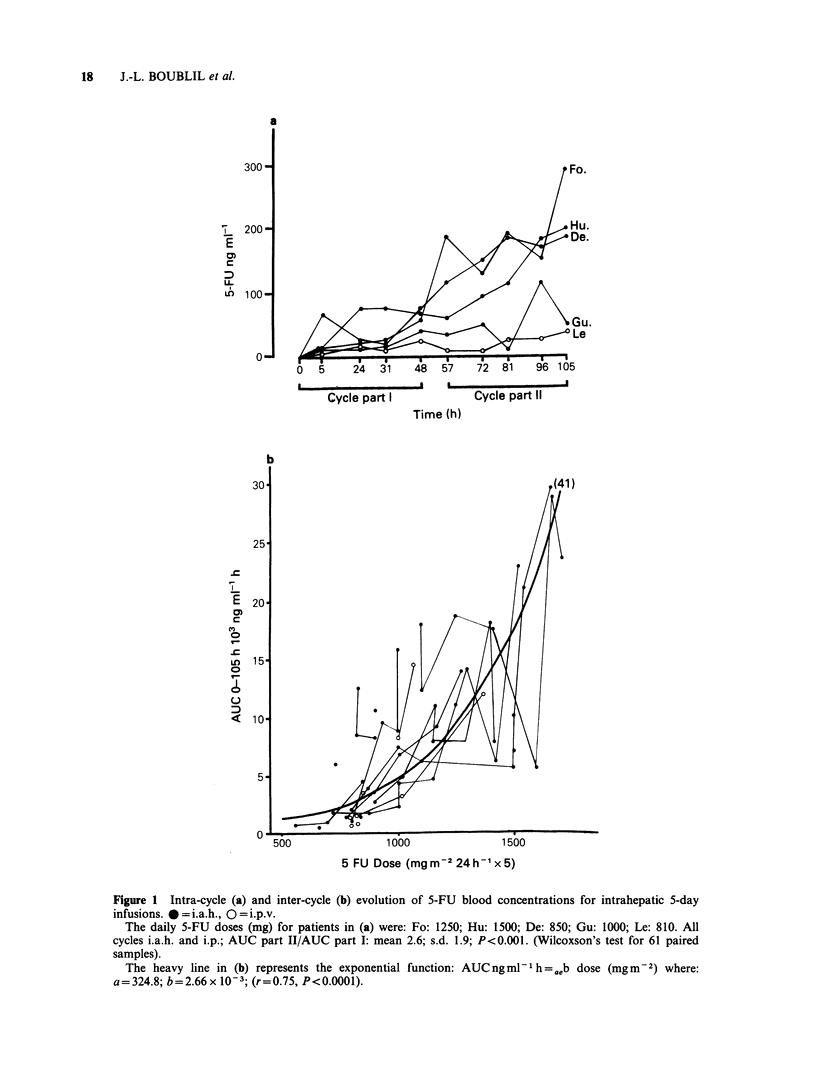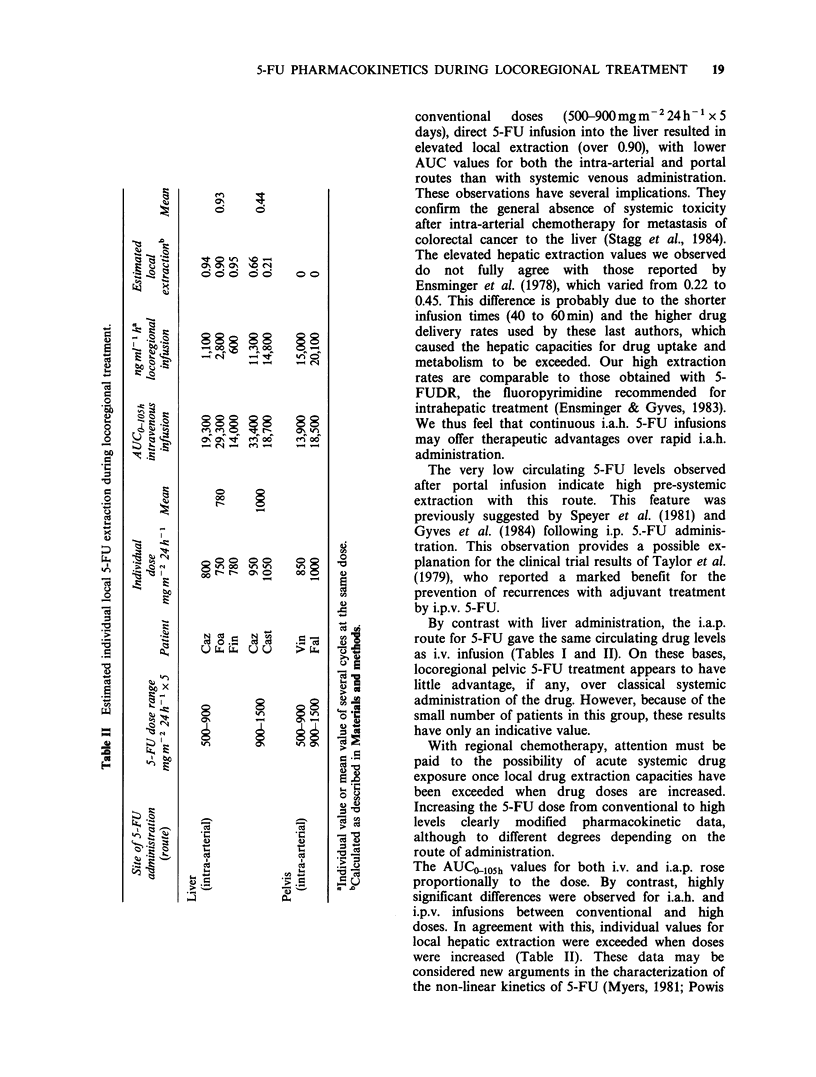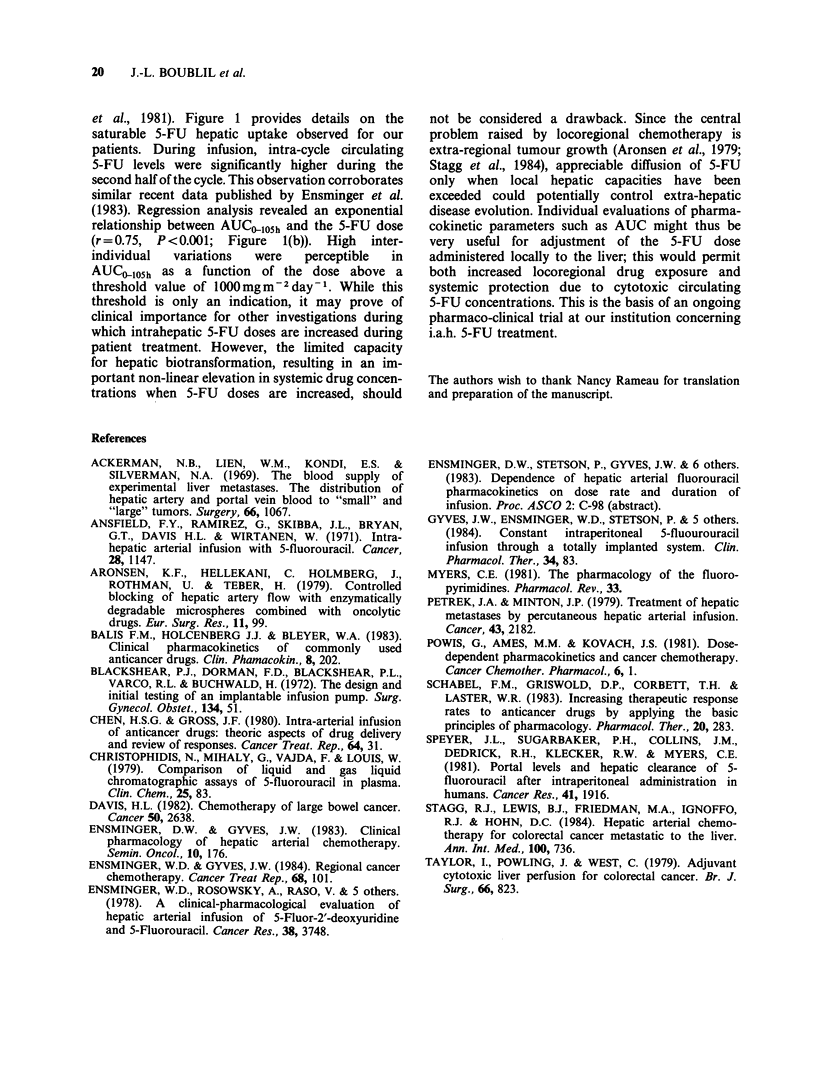# Continuous 5-day regional chemotherapy by 5-fluorouracil in colon carcinoma: pharmacokinetic evaluation.

**DOI:** 10.1038/bjc.1985.142

**Published:** 1985-07

**Authors:** J. L. Boublil, G. Milano, R. Khater, J. Bourry, A. Thyss, J. N. Bruneton, N. Renée, C. Philip, M. Namer

## Abstract

Eighteen patients with liver metastasis or locoregional recurrence of colon carcinoma received locoregional treatment by continuous 5-day infusions of 5-FU. 5-FU blood levels were measured by HPLC every day of the cycle at 8 am and 5 pm for a total of 87 cycles. Twelve patients were given the drug by an intra-arterial hepatic (i.a.h.) route, 3 by the portal vein (i.p.v.) and 3 by an intra-arterial pelvic (i.a.p.) route. These three routes were compared in respect of their relative pre-systemic drug uptake and the effect of dose escalation. Both the i.a.h. and i.p.v. routes, but not the i.a.p. route, resulted in a significant reduction in AUC 0-105 h compared to the i.v. route at the same dose range. Increasing the dose led to a modification in circulating 5-FU levels proportional to the dose for the i.v. and i.a.p. routes. By contrast, for the i.a.h. and i.p.v. routes, systemic drug delivery was significantly elevated, out of proportion with the dose, indicating a saturable process. For the i.a.h. route, increasing the 5-FU dose from 780 to 1000 mg m-2 day-1 caused a drop in hepatic extraction from 0.93 (0.90-0.95) to 0.44 (0.21-0.66). Liver saturation mechanisms were also evidenced by a mean increase of 2.6 times for the circulating drug level during the second part of the cycle as compared to the first part (P less than 0.001). The evolution of 5-FU AUC 0-105 h as a function of the dose was exponential (r = 0.75, P less than 0.001). Local extraction consecutive to i.a.p. was non-existent, implying that this route of drug administration has no potential advantage over classical i.v. infusion.


*Br. J. Cancer* (1985), **52**, 15–20

### Continuous 5-day regional chemotherapy by 5-fluorouracil in colon carcinoma: pharmacokinetic evaluation

J.-Louis Boublil[1], G. Milano[2], R. Khater[1], J. Bourry[1], A. Thyss[1], J.-N. Bruneton[1], N. Renée[2], C. Philip[3] & M. Namer[1]

*Centre Antoine Lacassagne: [1]Department Medical Oncology; [2]Pharmacokinetics Unit; and [3]Statistics Unit, 36 Voie Romaine, 06054 Nice Cedex, France.*

**Summary** Eighteen patients with liver metastasis or locoregional recurrence of colon carcinoma received locoregional treatment by continuous 5-day infusions of 5-FU. 5-FU blood levels were measured by HPLC every day of the cycle at 8 am and 5 pm for a total of 87 cycles. Twelve patients were given the drug by an intra-arterial hepatic (i.a.h.) route, 3 by the portal vein (i.p.v.) and 3 by an intra-arterial pelvic (i.a.p.) route. These three routes were compared in respect of their relative pre-systemic drug uptake and the effect of dose escalation. Both the i.a.h. and i.p.v. routes, but not the i.a.p. route, resulted in a significant reduction in $AUC_{0-105h}$ compared to the i.v. route at the same dose range. Increasing the dose led to a modification in circulating 5-FU levels proportional to the dose for the i.v. and i.a.p. routes. By contrast, for the i.a.h. and i.p.v. routes, systemic drug delivery was significantly elevated, out of proportion with the dose, indicating a saturable process. For the i.a.h. route, increasing the 5-FU dose from 780 to 1000 mg m$^{-2}$ day$^{-1}$ caused a drop in hepatic extraction from 0.93 (0.90–0.95) to 0.44 (0.21–0.66). Liver saturation mechanisms were also evidenced by a mean increase of 2.6 times for the circulating drug level during the second part of the cycle as compared to the first part ($P<0.001$). The evolution of 5-FU $AUC_{0-105h}$ as a function of the dose was exponential ($r=0.75$, $P<0.001$). Local extraction consecutive to i.a.p. was non-existent, implying that this route of drug administration has no potential advantage over classical i.v. infusion.

Locoregional chemotherapy has the dual aim of increasing the exposure of tumor-bearing areas to drugs while partially reducing the cytotoxic effects on the patient's healthy tissues (Ansfield *et al.*, 1971; Ensminger *et al.*, (1978). The recent interest shown in this therapeutic approach can be explained by better knowledge about pharmacokinetics, allowing more objective and rational use of drugs (Balis *et al.*, 1983; Schabel *et al.*, 1983) and by biotechnological progress in the field of implantable pumps (Blackshear *et al.*, 1972) permitting chemotherapy infusions on an ambulatory basis.

Fluoropyrimidines represent the predominant class of drugs used for locoregional chemotherapy of colorectal cancer (Davis, 1982). Long clinical experience has been gained with chemotherapy protocols administered by an intra-arterial hepatic (i.a.h.) route (Ansfield *et al.*, 1971; Stagg *et al.*, 1984; Ensminer *et al.*, 1978). Use of the portal vein appears advisable for small liver metastases (Ackerman *et al.*, 1969). Immediate post-surgery infusion of 5-flourouracil (5-FU) in the portal vein has recently been shown to significantly reduce the rate of disease recurrence at two years for Dukes' C lesions (Taylor *et al.*, 1979). Ensminger *et al.* (1978) have published detailed pharmacokinetic data concerning the i.a.h. administration of 5-FU, but for short infusions (40 to 60 min) at dose rates 10–100 times higher than those usually used. Their conclusions are thus difficult to extrapolate to continuous 5-day infusions which are the most commonly used regimens today (Petrek *et al.*, 1979; Stagg *et al.*, 1984). Only indirect pharmacokinetic data are available for intra-portal (i.p.v.) 5-FU infusion. However, Speyer *et al.* (1981) and Gyves *et al.* (1984), on the basis of i.p. 5-FU administration, suggested that considerable hepatic 5-FU extraction might occur through portal vein circulation. No pharmacokinetic evaluations have been published to date concerning intra-arterial pelvic (i.a.p.) 5-FU treatment aimed at better control of locoregional recurrences of colorectal cancer.

This study present pharmacokinetic data collected for 18 patients with liver metastasis or locoregional recurrence of colorectal cancer treated by continuous 5-day regional infusion of 5-FU. (i.a.h.), i.p.v. and i.a.p. routes were compared at increasing drug doses.

### Materials and methods

### *Patients*

All 18 patients (13 male, 5 female) had histologically confirmed colorectal cancer. Mean patients age was 65 years (range 57–78). Three of the

Correspondence: G. Milano.
Received 19 November 1984; and in revised form 5 March 1985.

© The Macmillan Press Ltd., 1985

patients had only locoregional disease recurrence; the other 15 had liver metastasis but no pelvic recurrence. Twelve patients were treated by the i.a.h. route, 3 by the i.p.v. route, and 3 by the i.a.p. route. Criteria for inclusion in the study were: estimated survival of $>3$ months, performance status $\leq 2$ (ECOG), no ascites neutrophilic polynuclear cells $>2500\,mm^{-3}$, platelets $>100{,}000\,mm^{-3}$, and total bilirubin $<1.5\,mg\,dl^{-1}$.

*Catheter insertion*

Catheters were inserted using an axillary or femoral artery route under local anesthesia. The tip of the catheter was placed in the hypogastric artery for patients with pelvic recurrences and in the hepatic artery for patients with liver metastasis. Three patients underwent surgery for insertion of a catheter in the portal vein, via the ombilical vein. Angiographic controls were performed systematically, during each cycle, prior to the start of 5-FU infusion.

*Chemotherapy protocols*

5-FU (Roche, France) was given by continuous 5-day infusion at doses varying from 500 to $1650\,mg\,m^{-2}\,24\,h^{-1}$. The daily dose of 5-FU was diluted in 2 l of 5% dextrose in water (D5W) using an external delivery pump (Fresenius, France). No other concurrent treatments were administered. Antiemetics were not used. The interval between the start of two successive cycles was 4 weeks. A total of 87 cycles was analyzed, 17 of which concerned 5-FU administered by a peripheral venous route (i.v.). In an attempt to simplify data presentation, 5-FU doses were classed in two groups: conventional doses: $500–900\,mg\,m^{-2}\,24\,h^{-1}$ and high doses: $900–1650\,mg\,m^{-2}\,24\,h^{-1}$.

*Pharmacokinetics*

Blood samples were obtained every day of each cycle at 8 am and 5 pm, i.e. at 0 h, 9 h, 24 h, 33 h, 48 h, 57 h, 72 h, 81 h, 96 h and 105 h. Samples (5 ml on EDTA tubes) were rapidly transferred to the laboratory. After centrifugation (10 min, 4°C, 2500 rpm), the supernatant was frozen at −20°C until analysis. Plasma 5-FU measurement was performed by HPLC (Christophidis *et al.*, 1979); the limit of sensitivity was $5\,ng\,ml^{-1}$. The day-to-day variation coefficient evaluated for 14 different analyses of a plasma spiked with $1000\,ng\,ml^{-1}$ was 4% (sd/mean × 100). The trapezoidal rule was used to compute the area under the curve (AUC) for the entire cycle, i.e. $AUC_{0-105\,h}$. Based on the theoretical considerations described by Chen & Gross (1980), the local extraction parameter was defined as follows:

$$\text{local extraction} = 1 - \left[\frac{AUC_{0-105\,h}R}{AUC_{0-105\,h}i.v.}\right]$$

where $AUC_{0-105h}i.v.$ = area under the curve after peripheral intravenous administration and $AUC_{0-105h}R$ = area under the curve after regional administration (i.a.h. or i.a.p. or i.p.v.) of the same dose as given by i.v.

This parameter was only evaluated for patients who received 5-FU i.v. infusions in cycles just before locoregional treatment.

### Results

Table I gives the respective values of the areas under the plasma concentration $x$ time curve ($AUC_{0-105h}$) for the different sites of locoregional 5-FU treatment. Data collected for i.v. infusions of 6 patients in the study have been shown as control values, corresponding to systemic circulating 5-FU levels when the drug is administered directly into the general circulation. Intra-hepatic (i.a.h. and i.p.v.) administration of 5-FU appears to significantly reduce the circulating levels of the drug as compared to the values observed following i.v. infusion. By contrast, no significant change was noted in $AUC_{0-105h}$ between i.a.p. and i.v. infusions.

Increasing the 5-FU dose led to different modifications in pharmacokinetics, depending on the route of drug administration. Thus, for i.v. and i.a.p., the mean 5-FU circulating levels rose proportionately with the dose increase. By contrast, the $AUC_{0-105h}$ values for i.a.h. and i.p.v. increased out of proportion to dose increases, and significant differences were noted between conventional and high 5-FU doses. Figure 1 details the changes observed in the i.a.h. and i.p.v. groups for both intra- and inter-cycle drug levels. Circulating 5-FU blood levels were 2.6 times higher during the second half of the cycle than during the first half ($P<0.001$); Figure 1(a) presents the typical profile for 5 patients. A saturation mechanism is also perceptible when the evolution of $AUC_{0-105h}$ is considered as a function of the dose (Figure 1(b)): 5-FU $AUC_{0-105h}$ is linked to the dose by an exponential function ($r=0.75$, $P<0.001$). A dose of $1000\,mg\,m^{-2}\,day^{-1}$ appears to be the critical threshold value above which wide inter-patient variations become patent.

Table II presents the estimated local extraction values for patients who received 5-FU by i.v. infusion prior to locoregional treatment at the same dose. With conventional doses, hepatic extraction

**Table I** Systemic 5-FU exposure after i.v. and locoregional administration.

| | *No. of patients*[a] | *No. of cycles* | *Mean 5-FU doses* $mg\ 24^{-1}\ h$ *(s.d.)* | $AUC_{0-105h}$ $ng\ ml^{-1}\ h$ *mean (s.d.)* | *Statistical analysis* | |
|---|---|---|---|---|---|---|
| | | | | | *See footnote*[b] | *See footnote*[c] |
| *Intravenous 5-FU* | | | | | | |
| conventional doses[d] | 5 | 13 | 780 (93) | 14,000 (8000) | $t=1.9$ | |
| high doses | 4 | 4 | 954 (27) | 22,400 (7300) | $df=15$ | |
| ratio[e] | | | 1.22 | 1.60 | NS | |
| *Intra-arterial hepatic 5-FU* | | | | | | |
| conventional doses | 11 | 18 | 804 (96) | 4030 (3600) | | $t=3.93$ |
| | | | | | $t=4.18$ | $df=29$ |
| | | | | | $df=50$ | $P<0.001$ |
| | | | | | $P<0.001$ | |
| high doses | 7 | 34 | 1280 (230) | 12,800 (8400) | | $t=2.17$ |
| | | | | | | $df=36$ |
| ratio | | | 1.59 | 3.78 | | $0.02<P<0.05$ |
| *Intra-portal 5-FU* | | | | | | |
| conventional doses | 3 | 5 | 804 (16) | 1560 (1110) | | $t=3.06$ |
| | | | | | | $df=16$ |
| | | | | | $t=3.43$ | $0.001<P<0.01$ |
| | | | | | $df=7$ | |
| high doses | 2 | 4 | 1110 (163) | 9550 (5150) | $0.01<P<0.02$ | $t=2.88$ |
| | | | | | | $df=6$ |
| ratio | | | 1.38 | 6.12 | | $0.02<P<0.05$ |
| *Intra-arterial pelvic 5-FU* | | | | | | |
| conventional doses | 2 | 5 | 677 (99) | 16,400 (5100) | | $t=0.59$ |
| | | | | | | $df=16$ |
| | | | | | $t=0.89$ | NS |
| | | | | | $df=7$ | |
| high doses | 1 | 4 | 1058 (68) | 19,800 (5800) | NS | $t=0.58$ |
| | | | | | | $df=6$ |
| ratio | | | 1.56 | 1.21 | | NS |

[a]Certain patients were in both 5-FU dose groups.
[b]Student's $t$-test for comparison of AUC between conventional and high doses.
[c]Student's $t$-test for comparison of AUC with corresponding i.v. control group.
[d]Conventional and high doses as defined in **Materials and methods.**
[e]Ratios of mean values determined at high doses/conventional doses.

was high (mean 0.93) for the 3 patients evaluated; by contrast, increasing doses led to a drop in hepatic uptake (0.21 and 0.66 for the 2 patients evaluated). In comparison, and in confirmation of the data in Table I, i.a.p. administration of 5-FU did not involve pre-systemic extraction.

### Discussion

Existence of a dose/response relationship is one of the criteria for selection of anticancer agents for locoregional treatment (Ensminger & Gyves, 1984). Experimentally, 5-FU appears to fulfil this condition (Schabel *et al.*, 1983). On this basis, increasing local drug exposure can reasonably be expected to have a high probability of causing more quantitative regression than an equivalent dose administered by a venous route. The data in the present study were collected during the normal course of different types of locoregional treatment with 5-FU for patients with colorectal cancer. With

**Figure 1** Intra-cycle (**a**) and inter-cycle (**b**) evolution of 5-FU blood concentrations for intrahepatic 5-day infusions. ● = i.a.h., ○ = i.p.v.

The daily 5-FU doses (mg) for patients in (**a**) were: Fo: 1250; Hu: 1500; De: 850; Gu: 1000; Le: 810. All cycles i.a.h. and i.p.; AUC part II/AUC part I: mean 2.6; s.d. 1.9; $P<0.001$. (Wilcoxson's test for 61 paired samples).

The heavy line in (**b**) represents the exponential function: AUC ng ml$^{-1}$ h $= {}_{a}e^{b}$ dose (mg m$^{-2}$) where: $a=324.8$; $b=2.66\times10^{-3}$; ($r=0.75$, $P<0.0001$).

**Table II** Estimated individual local 5-FU extraction during locoregional treatment.

| *Site of 5-FU administration (route)* | *5-FU dose range* $mg\,m^{-2}\,24\,h^{-1}\times 5$ | *Patient* | *Individual dose* $mg\,m^{-2}\,24\,h^{-1}$ | *Mean* | $AUC_{0-105h}$ *intravenous infusion* | $ng\,ml^{-1}\,h$[a] *locoregional infusion* | *Estimated local extraction*[b] | *Mean* |
|---|---|---|---|---|---|---|---|---|
| Liver (intra-arterial) | 500–900 | Caz | 800 | | 19,300 | 1,100 | 0.94 | |
| | | Foa | 750 | 780 | 29,300 | 2,800 | 0.90 | 0.93 |
| | | Fin | 780 | | 14,000 | 600 | 0.95 | |
| | 900–1500 | Caz | 950 | 1000 | 33,400 | 11,300 | 0.66 | 0.44 |
| | | Cast | 1050 | | 18,700 | 14,800 | 0.21 | |
| Pelvis (intra-arterial) | 500–900 | Vin | 850 | | 13,900 | 15,000 | 0 | |
| | 900–1500 | Fal | 1000 | | 18,500 | 20,100 | 0 | |

[a]Individual value or mean value of several cycles at the same dose.

[b]Calculated as described in **Materials and methods**.

conventional doses ($500–900\,mg\,m^{-2}\,24\,h^{-1}\times 5$ days), direct 5-FU infusion into the liver resulted in elevated local extraction (over 0.90), with lower AUC values for both the intra-arterial and portal routes than with systemic venous administration. These observations have several implications. They confirm the general absence of systemic toxicity after intra-arterial chemotherapy for metastasis of colorectal cancer to the liver (Stagg *et al.*, 1984). The elevated hepatic extraction values we observed do not fully agree with those reported by Ensminger *et al.* (1978), which varied from 0.22 to 0.45. This difference is probably due to the shorter infusion times (40 to 60 min) and the higher drug delivery rates used by these last authors, which caused the hepatic capacities for drug uptake and metabolism to be exceeded. Our high extraction rates are comparable to those obtained with 5-FUDR, the fluoropyrimidine recommended for intrahepatic treatment (Ensminger & Gyves, 1983). We thus feel that continuous i.a.h. 5-FU infusions may offer therapeutic advantages over rapid i.a.h. administration.

The very low circulating 5-FU levels observed after portal infusion indicate high pre-systemic extraction with this route. This feature was previously suggested by Speyer *et al.* (1981) and Gyves *et al.* (1984) following i.p. 5.-FU administration. This observation provides a possible explanation for the clinical trial results of Taylor *et al.* (1979), who reported a marked benefit for the prevention of recurrences with adjuvant treatment by i.p.v. 5-FU.

By contrast with liver administration, the i.a.p. route for 5-FU gave the same circulating drug levels as i.v. infusion (Tables I and II). On these bases, locoregional pelvic 5-FU treatment appears to have little advantage, if any, over classical systemic administration of the drug. However, because of the small number of patients in this group, these results have only an indicative value.

With regional chemotherapy, attention must be paid to the possibility of acute systemic drug exposure once local drug extraction capacities have been exceeded when drug doses are increased. Increasing the 5-FU dose from conventional to high levels clearly modified pharmacokinetic data, although to different degrees depending on the route of administration.

The $AUC_{0-105h}$ values for both i.v. and i.a.p. rose proportionally to the dose. By contrast, highly significant differences were observed for i.a.h. and i.p.v. infusions between conventional and high doses. In agreement with this, individual values for local hepatic extraction were exceeded when doses were increased (Table II). These data may be considered new arguments in the characterization of the non-linear kinetics of 5-FU (Myers, 1981; Powis

*et al.*, 1981). Figure 1 provides details on the saturable 5-FU hepatic uptake observed for our patients. During infusion, intra-cycle circulating 5-FU levels were significantly higher during the second half of the cycle. This observation corroborates similar recent data published by Ensminger *et al.* (1983). Regression analysis revealed an exponential relationship between $AUC_{0-105h}$ and the 5-FU dose ($r=0.75$, $P<0.001$; Figure 1(b)). High inter-individual variations were perceptible in $AUC_{0-105h}$ as a function of the dose above a threshold value of $1000\,mg\,m^{-2}\,day^{-1}$. While this threshold is only an indication, it may prove of clinical importance for other investigations during which intrahepatic 5-FU doses are increased during patient treatment. However, the limited capacity for hepatic biotransformation, resulting in an important non-linear elevation in systemic drug concentrations when 5-FU doses are increased, should not be considered a drawback. Since the central problem raised by locoregional chemotherapy is extra-regional tumour growth (Aronsen *et al.*, 1979; Stagg *et al.*, 1984), appreciable diffusion of 5-FU only when local hepatic capacities have been exceeded could potentially control extra-hepatic disease evolution. Individual evaluations of pharmacokinetic parameters such as AUC might thus be very useful for adjustment of the 5-FU dose administered locally to the liver; this would permit both increased locoregional drug exposure and systemic protection due to cytotoxic circulating 5-FU concentrations. This is the basis of an ongoing pharmaco-clinical trial at our institution concerning i.a.h. 5-FU treatment.

The authors wish to thank Nancy Rameau for translation and preparation of the manuscript.

### References

ACKERMAN, N.B., LIEN, W.M., KONDI, E.S. & SILVERMAN, N.A. (1969). The blood supply of experimental liver metastases. The distribution of hepatic artery and portal vein blood to "small" and "large" tumors. *Surgery*, **66**, 1067.

ANSFIELD, F.Y., RAMIREZ, G., SKIBBA, J.L., BRYAN, G.T., DAVIS H.L. & WIRTANEN, W. (1971). Intra-hepatic arterial infusion with 5-fluorouracil. *Cancer*, **28**, 1147.

ARONSEN, K.F., HELLEKANI, C. HOLMBERG, J., ROTHMAN, U. & TEBER, H. (1979). Controlled blocking of hepatic artery flow with enzymatically degradable microspheres combined with oncolytic drugs. *Eur. Surg. Res.*, **11**, 99.

BALIS F.M., HOLCENBERG J.J. & BLEYER, W.A. (1983). Clinical pharmacokinetics of commonly used anticancer drugs. *Clin. Phamacokin.*, **8**, 202.

BLACKSHEAR, P.J., DORMAN, F.D., BLACKSHEAR, P.L., VARCO, R.L. & BUCHWALD, H. (1972). The design and initial testing of an implantable infusion pump. *Surg. Gynecol. Obstet.*, **134**, 51.

CHEN, H.S.G. & GROSS, J.F. (1980). Intra-arterial infusion of anticancer drugs: theoric aspects of drug delivery and review of responses. *Cancer Treat. Rep.*, **64**, 31.

CHRISTOPHIDIS, N., MIHALY, G., VAJDA, F. & LOUIS, W. (1979). Comparison of liquid and gas liquid chromatographic assays of 5-fluorouracil in plasma. *Clin. Chem.*, **25**, 83.

DAVIS, H.L. (1982). Chemotherapy of large bowel cancer. *Cancer* **50**, 2638.

ENSMINGER, D.W. & GYVES, J.W. (1983). Clinical pharmacology of hepatic arterial chemotherapy. *Semin. Oncol.*, **10**, 176.

ENSMINGER, W.D. & GYVES, J.W. (1984). Regional cancer chemotherapy. *Cancer Treat Rep.*, **68**, 101.

ENSMINGER, W.D., ROSOWSKY, A., RASO, V. & 5 others. (1978). A clinical-pharmacological evaluation of hepatic arterial infusion of 5-Fluor-2′-deoxyuridine and 5-Fluorouracil. *Cancer Res.*, **38**, 3748.

ENSMINGER, D.W., STETSON, P., GYVES, J.W. & 6 others. (1983). Dependence of hepatic arterial fluorouracil pharmacokinetics on dose rate and duration of infusion. *Proc. ASCO* 2: C-98 (abstract).

GYVES, J.W., ENSMINGER, W.D., STETSON, P. & 5 others. (1984). Constant intraperitoneal 5-fluourouracil infusion through a totally implanted system. *Clin. Pharmacol. Ther.*, **34**, 83.

MYERS, C.E. (1981). The pharmacology of the fluoropyrimidines. *Pharmacol. Rev.*, **33.**

PETREK, J.A. & MINTON, J.P. (1979). Treatment of hepatic metastases by percutaneous hepatic arterial infusion. *Cancer*, **43**, 2182.

POWIS, G., AMES, M.M. & KOVACH, J.S. (1981). Dose-dependent pharmacokinetics and cancer chemotherapy. *Cancer Chemother. Pharmacol.*, **6**, 1.

SCHABEL, F.M., GRISWOLD, D.P., CORBETT, T.H. & LASTER, W.R. (1983). Increasing therapeutic response rates to anticancer drugs by applying the basic principles of pharmacology. *Pharmacol. Ther.*, **20**, 283.

SPEYER, J.L., SUGARBAKER, P.H., COLLINS, J.M., DEDRICK, R.H., KLECKER, R.W. & MYERS, C.E. (1981). Portal levels and hepatic clearance of 5-fluorouracil after intraperitoneal administration in humans. *Cancer Res.*, **41**, 1916.

STAGG, R.J., LEWIS, B.J., FRIEDMAN, M.A., IGNOFFO, R.J. & HOHN, D.C. (1984). Hepatic arterial chemotherapy for colorectal cancer metastatic to the liver. *Ann. Int. Med.*, **100**, 736.

TAYLOR, I., POWLING, J. & WEST, C. (1979). Adjuvant cytotoxic liver perfusion for colorectal cancer. *Br. J. Surg.*, **66**, 823.